# Cyclodextrins Initiated Ring-Opening Polymerization of Lactide Using 4-Dimethylaminopyridine (DMAP) as Catalyst: Study of DMAP/β-CD Inclusion Complex and Access to New Structures

**DOI:** 10.3390/molecules27031083

**Published:** 2022-02-06

**Authors:** Julie Meimoun, Yupin Phuphuak, Remi Miyamachi, Yong Miao, Marc Bria, Cyril Rousseau, Guilherme Nogueira, Andreia Valente, Audrey Favrelle-Huret, Philippe Zinck

**Affiliations:** 1 Univ. Lille, CNRS, Centrale Lille, Univ. Artois, UMR 8181—UCCS—Unité de Catalyse et Chimie du Solide, F-59000 Lille, France; julie.meimoun90@gmail.com (J.M.); yupinp@nu.ac.th (Y.P.); cherry.milky.853@gmail.com (R.M.); Yong.Miao@gnubiotics.com (Y.M.); guilherme.nogueira91@gmail.com (G.N.); 2 Department of Chemistry, Faculty of Science, Naresuan University, Phitsanulok 65000, Thailand; 3 Univ. Lille, CNRS, INRA, Centrale Lille, ENSCL, Univ. Artois, FR 2638—IMEC—Institut Michel-Eugène Chevreul, Pôle RMN, F-59000 Lille, France; marc.bria@univ-lille.fr; 4 Univ. Lille, CNRS, Centrale Lille, ENSCL, Univ. Artois, UMR 8181—UCCS—Unité de Catalyse et Chimie du Solide, F-62300 Lens, France; cyril.rousseau@univ-artois.fr; 5 Centro de Química Estrutural, Institute of Molecular Sciences and Departamento de Química e Bioquímica, Faculdade de Ciências, Universidade de Lisboa, Campo Grande, 1749-016 Lisboa, Portugal; amvalente@fc.ul.pt

**Keywords:** ring-opening polymerization, *rac*-lactide, polylactide, cyclodextrin, organocatalysis, DMAP, inclusion complex, polylactide carbohydrate conjugate

## Abstract

Cyclodextrins (CDs) are cyclic oligosaccharides used in many fields. Grafting polymers onto CDs enables new structures and applications to be obtained. Polylactide (PLA) is a biobased, biocompatible aliphatic polyester that can be grafted onto CDs by -OH-initiated ring-opening polymerization. Using 4-dimethylaminopyridine (DMAP) as an organocatalyst, a quantitative functionalization is reached on native α-, β-, γ- and 2,3-dimethyl- β-cyclodextrins. Narrow molecular weight distributions are obtained with the native CDs (dispersity < 1.1). The DMAP/β-CD combination is used as a case study, and the formation of an inclusion complex (1/1) is shown for the first time in the literature, which is fully characterized by NMR. The inclusion of DMAP into the cavity occurs via the secondary rim of the β-CD and the association constant (K_a_) is estimated to be 88.2 M^−1^. Its use as an initiator for ring-opening polymerization leads to a partial functionalization efficiency, and thus a more hydrophilic β-CD-PLA conjugate than that obtained starting from native β-CD. Polymerization results including also the use of the adamantane/β-CD inclusion complex as an initiator suggest that inclusion of the DMAP catalyst into the CD may not occur during polymerization reactions. *Rac*-lactide does not form an inclusion complex with β-CD.

## 1. Introduction

Cyclodextrins (CDs) are cyclic oligosaccharides produced from starch by enzymatic degradation. The most well-known CDs are composed of six (α-CD), seven (β-CD) or eight (γ-CD) α-D-(1→4) glucopyranoside moieties. The hydrophobic, size-selective toroidal cavity with C-6 primary hydroxyl groups on the narrow rim and C-2 and C-3 secondary hydroxyl groups on the wider rim is well known to incorporate a large range of hydrophobic molecules. Due to this capacity to form inclusion complexes and the properties of amphiphilic structures, CDs are very interesting for many industrial applications such as in chemistry, pharmaceutical science and chromatography, and in food, cosmetic, textile and environmental fields [1,2,3,4,5,6,7].

Over many decades, several studies have been carried out in order to selectively modify native cyclodextrins in chemical [8,9] and enzymatical [10,11,12] ways. Two main factors are to be considered in CD chemistry: the nucleophilicity of hydroxyl groups and the capacity of CDs to form inclusion complexes with the reagents used. Whatever modification is made, it will take place on the hydroxyl groups. Thus, the OH6 groups are the most basic, the most accessible and the most reactive. Those in position 2 are the most acidic (pK_a_ = 12.2) while those in position 3 are the least reactive and the least accessible.

Among modified CDs, cyclodextrin-based polymer materials have numerous applications in the biomedical and pharmaceutical fields [13], and also for dye and fragrance [14] or as additives for improving the properties of biobased polymers [15]. Among these materials, aliphatic polyester functionalized cyclodextrins are particularly attractive, as the remaining object is fully biocompatible, and in the case of polylactide, fully biobased. Different synthetic strategies are available to access these macromolecular objects, such as direct coupling, or the use of the hydroxyl groups of cyclodextrin as initiators for the ring-opening polymerization (ROP) of cyclic esters [16,17]. This can be done with or without the use of a catalyst. Regarding the latter, Harada et al. reported the cyclodextrin initiated polymerization of lactones in bulk in the absence of any other co-catalyst [18,19]. Number-average degrees of polymerization up to 10 were obtained, with up to 1/3 initiating OH groups. The resulting oligoester arms were found to be covalently linked to the C2 carbon of the glucopyranose unit. Later, it was shown that the pressure can increase the activity of the bulk polymerization of ε-caprolactone initiated by wet β-CD [20].

Lactide (LA) could also be opened by β-cyclodextrin in dimethylformamide (DMF) without a co-catalyst, leading to a number-average degree of polymerization around 2 and a degree of substitution of 1.5, the oligolactide arms being grafted on the C6 carbon of the cyclodextrin [21]. In the same frame, Peptu et al. reported the green synthesis of oligolactide derivatives based on native α-, β- and γ- CD, in which CDs play the role of both initiator and catalyst in bulk for 72 h [22]. Mass spectrometry, NMR spectroscopy and reversed-phase liquid chromatography were used in order to determine the structure of the final products, composed of unreacted LA monomer, free CDs and polylactide (PLA) homopolymer in addition to CD-PLA conjugate. Moreover, the reaction was carried out selectively on the primary rim of CDs. CDs are linked with more than one oligolactate chain per CD molecule. The authors showed that the reactivity of the CDs was different for the ROP of LA, with the highest reactivity for the β-CD and a lower reactivity for the γ-CD.

**Figure 1:**
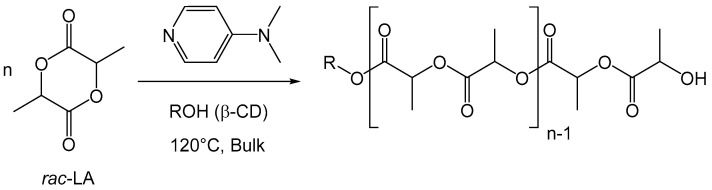
DMAP initiated ring-opening polymerization of *rac*-lactide initiated by β-CD.

If the of the absence of catalyst in these reactions is noteworthy, a more controlled character may be an advancement in the field, together with the access to polyester arms of higher degrees of polymerization and a full functionalization of the cyclodextrin involving all OH groups. We reported the first example of a fully functionalized polyester-cyclodextrin conjugate using a 4-dimethylaminopyridine (DMAP) catalyzed ring-opening polymerization of lactide (Figure 1) [23]. The number-average degree of polymerization could be controlled by the monomer/OH ratio, and the reaction proceeds without solvent, in bulk.

**Figure 2:**
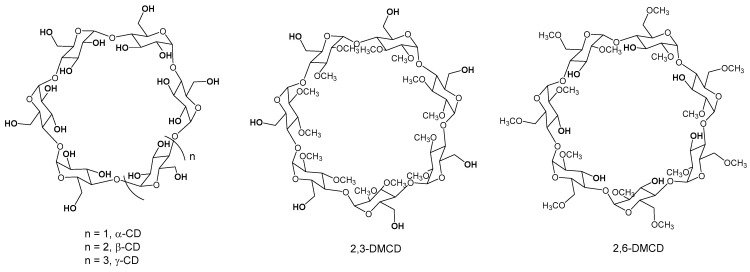
Structures of the different cyclodextrins used as initiators in this study.

In the present work, we have formed for the first time and studied a DMAP/β-CD inclusion complex, including its application to polymerization reactions. We further extended for the first time the DMAP catalyzed ring-opening polymerization of lactide to native α- and γ-cyclodextrins as co-initiators, as well as to the partially methylated heptakis-(2,3-di-*O*-methyl)-β-cyclodextrin (2,3-DMCD) and heptakis-(2,6-di-*O*-methyl)-β-cyclodextrin (2,6-DMCD) (see Figure 2), yielding several cyclodextrin-polylactide conjugates that had not been reported in the literature so far.

## 2. Results and Discussion

### 2.1. Ability of DMAP and Rac-Lactide to Form an Inclusion Complex with β-CD

The ability of DMAP and *rac*-lactide to form an inclusion complex with β-CD was first assessed via NMR. Indeed, NMR is a technique commonly used to demonstrate the formation of an inclusion complex between a guest molecule and the hydrophobic cavity of cyclodextrins as it provides both qualitative (highlighting the inclusion) and quantitative information (determining the stoichiometry and association constant). D_2_O was chosen as the solvent, as DMSO can enter into the cavity of the CD.

**Figure 3:**
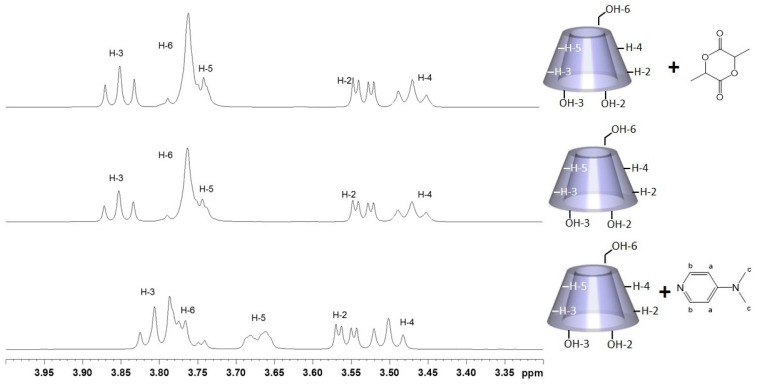
^1^H NMR model study of 1:1 DMAP/β-CD mixture (bottom), β-CD (middle) and 1:1 *rac*-LA/β-CD mixture (top) in D_2_O (300 MHz, 298 K, zoom between 3.3 and 4 ppm).

The (1:1) DMAP/β-CD mixture, β-CD and the (1:1) *rac*-LA/β-CD mixture were analyzed by ^1^H NMR. The three spectra are shown in Figure 3. We observed that the signals of H-3 and H-5 of β-CD in the DMAP/β-CD mixture (Figure 3, bottom) shifted compared with the β-CD itself (Figure 3, middle), while no important shift in the signals of β-CD in the *rac*-LA/β-CD mixture could be observed (Figure 3, top). This evidence of interaction between DMAP and β-CD suggests that DMAP may be included in the cavity of β-CD, whereas this is not the case for *rac*-LA.

In order to evidence the formation of the DMAP/β-CD inclusion complex and to characterize it, we conducted a more extensive NMR study. In solution, the inclusion of DMAP in the β-CD cavity is evidenced by the change in chemical shifts of some of the guest and host protons, in comparison with the chemical shifts of the same protons in free components (see Table 1; the full spectra are given in the SI Section Figure S1). As expected, we observed significant shifts of the H-3 and H-5 protons located inside the cavity of β-CD (host molecule) and the H-a proton of DMAP (guest molecule) and to a lesser extent the H-6 proton of β-CD and H-c proton of DMAP. This clearly shows the inclusion of DMAP inside the cavity of the β-CD.

As the size of DMAP and lactide are relatively close (in the order of 4–5 Å), the non-inclusion of lactide in the cavity of β-CD does not seem to be related to a size limitation. This could be explained by the fact that CDs are able to form non-inclusion complexes with certain organic molecules because the OH present on the external surface of the CD can form hydrogen bonds with these molecules. This contrasts with the ability of lactones to form inclusion complexes with cyclodextrins, such as the ε-caprolactone/β-CD inclusion complex [24]. However, the inclusion of nitrogenous heterocyclic bases such as, e.g., adenine [25], bipyridines [26] or α-aminopyridine [27] into β-CD has been reported in the literature. As far as we know, inclusion complexes between DMAP and β-cyclodextrin have never been reported in the literature. We decided thus to determine their stoichiometry and geometry, as well as the association constant.

### 2.2. Determination of the DMAP/β-CD Inclusion Complex Stoichiometry by the Job Plots Method

**Figure 4:**
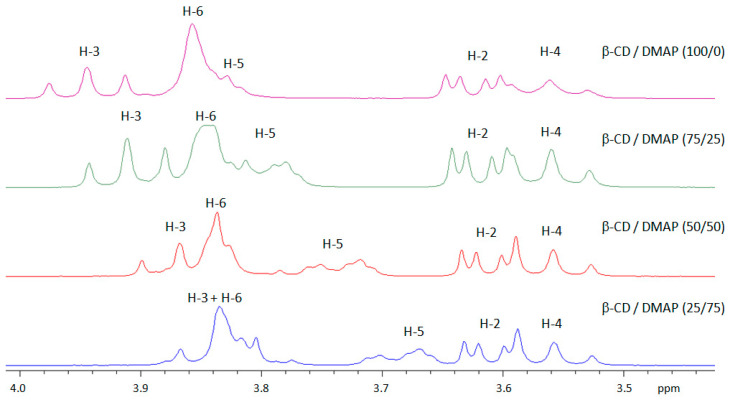
^1^H NMR spectra of β-CD and β-CD/DMAP complex at different ratios (D_2_O, 300 MHz, 298 K, total concentration [β-CD] + [DMAP] = 10 mM, zoom between 3.5–4 ppm).

The aforementioned ^1^H NMR evidence allows us to use the continuous variation method (i.e., Job plots method) to establish the stoichiometry of the formed inclusion complex. This method is based on the induced chemical shift variation, Δδ, which is directly related to the concentration of the complex and therefore a function of the molar proportion of the guest molecule, which makes it possible to determine the stoichiometry by a simple experimental plot. Δδ is defined as the chemical shift difference between the free and complexed substrate [28,29]. By varying the [β-CD]/[DMAP] ratio, the cyclodextrin protons located inside the cavity (H-3 and H-5) shifted considerably (Figure 4), confirming the interaction between DMAP and the inside cavity of β-CD.

**Figure 5:**
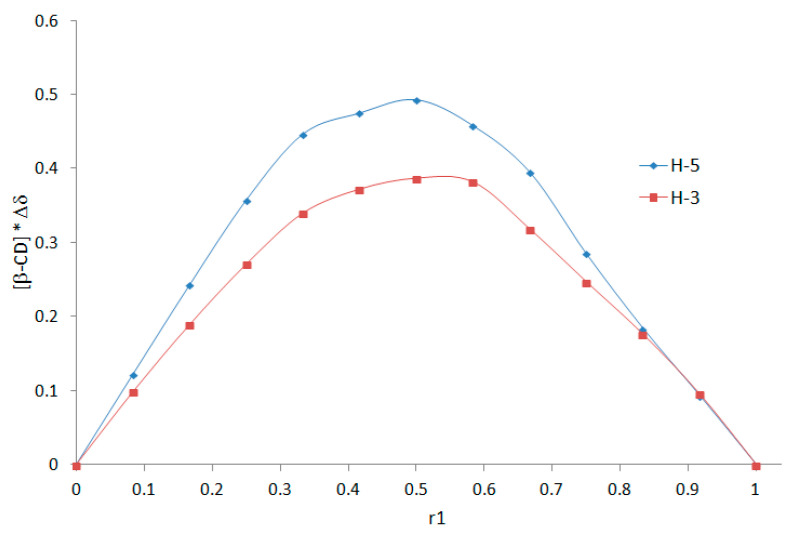

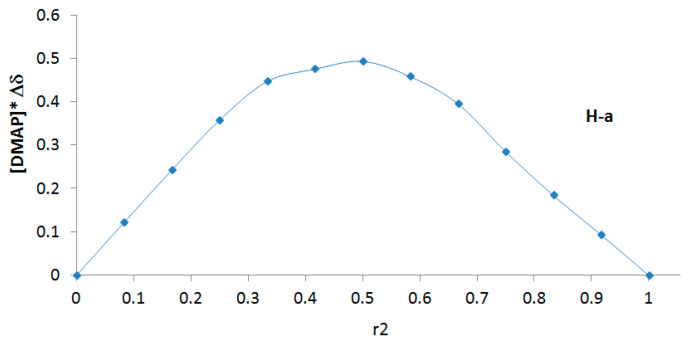
Job’s plots—chemical shift variation of the protons H-3, H-5, H-a for DMAP/β-CD system at different concentrations.

The chemical shifts of representative protons of β-CD (H-3, H-5) and DMAP (H-a) were quantified by ^1^H NMR via the following equation: Δδ = δ_free_ − δ_obs_ (chemical shift difference between the free and complexed substrate). If Δδ * [H] (host) or Δδ * [G] (guest) is plotted as a function r_1_ = [H]/([H] + [G]) or r_2_ = [G]/([H] + [G]) (Job’s plot), its maximum value will occur at r_1_ or r_2_. The different curves (represented in Figure 5) presented a maximum at 0.5 (in all cases), characteristic of a complex with 1/1 stoichiometry.

### 2.3. Characterization of the DMAP/β-CD Inclusion Complex Geometry

**Figure 6:**
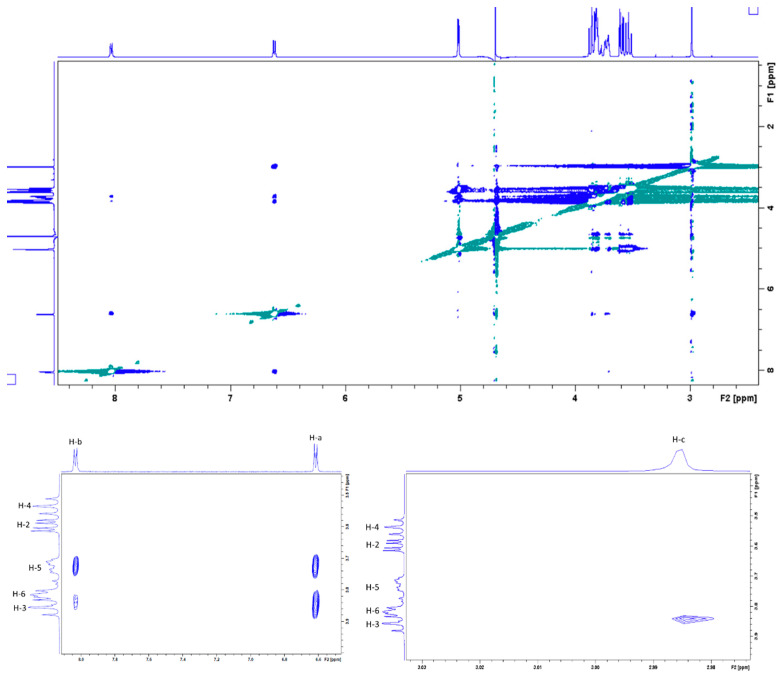
ROESY ^1^H NMR spectrum of DMAP/β-CD complex (D_2_O, 400 MHz, 300 K, [DMAP] = [β-CD] = 3.7 mM. Top: full spectrum. Bottom: zooms on correlation between H-a, H-b, H-3 and H-5 (**left**) and between H-c and H-3 (**right**).

**Figure 7:**
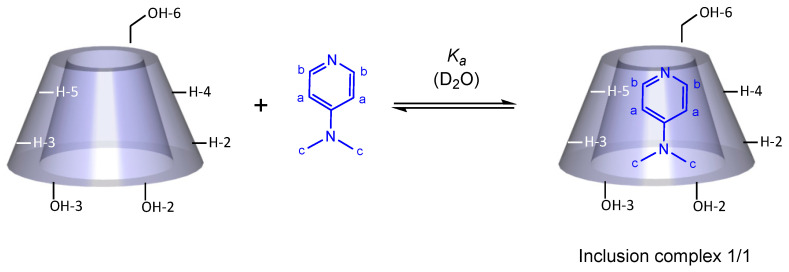
Scheme of the proposed representation of the DMAP inclusion in the hydrophobic β-CD cavity.

In order to obtain more information about the inclusion mode of the DMAP in the β-CD cavity, a 2D ROESY ^1^H NMR spectrum was acquired. Several intermolecular cross-peaks were observed between H-3 and H-5 protons of β-CD and H-a, H-b and H-c of DMAP, proving the complete inclusion of DMAP in the CD cavity (Figure 6). H-a correlated with H-3 and H-5, H-b with H-5 and to lesser extent with H-3, and H-c with H-3. According to these observations, the geometrical structure of the DMAP/β-CD inclusion complex, having 1/1 stoichiometry, is schematically presented in Figure 7.

### 2.4. Estimation of the Association Constant (K_a_) of DMAP/β-CD Inclusion Complex

In order to determine the strength of the intermolecular binding between DMAP and β-CD, approximations of the association constant *K*_a_ and Δδ_c_ (chemical shift difference between the free and completely complexed substrate) were determined from the Benesi–Hildebrand method via the following Equation [28]: 1/Δδ_obs_ = 1/(*K*_a_ * Δδ_c_ *[β-CD]) + 1/Δδ_c_, where Δδ_obs_ (defined by δ_free_ − δ_obs_) was determined by ^1^H NMR.

**Figure 8:**
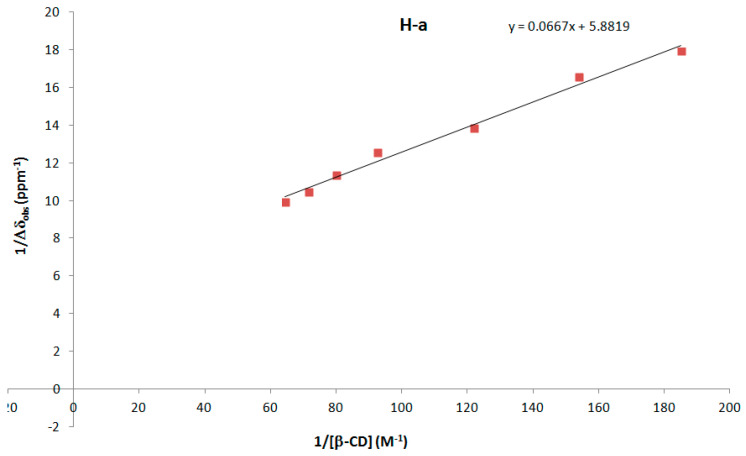
1/Δδ_obs_ as a function of 1/[β-CD] for the DMAP/β-CD complex.

The spectrum of DMAP alone was also recorded to determine δ_free_ (see SI Figure S1). Using the chemical shift changes of the H-a (DMAP) proton, the curve 1/Δδ_obs_ as a function of 1/[β-CD] was drawn (Figure 8). From the linear equation of the curve, Δδ_c_ = 1/5.8819 = 0.17 ppm and *K*_a_ = 5.8819/0.0667 = 88.2 M^−1^ were calculated.

### 2.5. Polymerization of Rac-Lactide Using the DMAP/β-CD Inclusion Complex as the Initiator

Based on the model study, we decided to synthesize the inclusion complex DMAP/β-CD by applying the synthesis in water, as reported for other similar inclusion complexes [26,27,30] (see experimental section). Entries representative of the polymerization of *rac*-lactide mediated by the inclusion complex are presented in Table 2. The DMAP/β-CD complex used alone led to very poor polymerization performances in standard conditions (entry 1). In combination with DMAP, and for a monomer/initiator ratio of 10, the activity obtained (entry 2) is similar to that observed using native β-CD as the initiator (entry 3), with almost full conversion in 30 min. If the dispersity of the molecular weight distribution obtained around 1.1 is also similar, the initiation efficiency is not quantitative for the inclusion complex, in contrast to the native carbohydrate. This may be ascribed to the interaction of hydroxyl groups with the included DMAP or a steric hindrance induced by the proximity of the DMAP. This finding suggests that (i) from a mechanistic point of view, the DMAP/β-CD inclusion complex is probably not formed in the course of the polymerization of *rac*-lactide initiated by the native cyclodextrin and catalyzed by DMAP. To obtain additional insights, the polymerization was further conducted starting from an adamantane/β-CD inclusion complex (entry 6). As the adamantane guest is strongly included in the cavity of the β-CD, it is expected that the DMAP/β-CD inclusion complex may not not formed. In these conditions, the polymerization of *rac*-lactide still occurs, yet with a lower activity. This shows that the polymerization can proceed without inclusion of DMAP into the cavity of the CD. (ii) From a macromolecular engineering perspective, this is interesting as it provides access to an alternative, more hydrophilic cyclodextrin polylactide conjugate microstructure, as ca. 1/3 of the hydroxyl functions of the cyclodextrin did not react. Note that the initiation efficiency is not affected in an important manner by the monomer/OH ratio, as shown in entries 4 and 5 for ratios of 2 and 20.

### 2.6. Polymerization of Rac-Lactide Using Other Native and O-Methylated Cyclodextrins as Initiators

We finally assessed the DMAP catalyzed ring-opening polymerization of *rac*-lactide initiated by other cyclodextrins, namely the native α- and γ-CD as well as the partially methylated heptakis-(2,3-di-*O*-methyl)-β-cyclodextrin (2,3-DMCD) and heptakis-(2,6-di-*O*-methyl)-β-cyclodextrin (2,6-DMCD) (see Figure 2). Using α-CD as the initiator, the polymerization is quantitative, with dispersities < 1.1 (entries 7–8). The initiation efficiency increases with the monomer/OH ratio, reaching a quantitative value for a ratio of 30. The process is also well suited for the synthesis of γ-CD polylactide conjugates, with almost quantitative reactions in 10 to 30 min (entries 9–10) and good dispersities (1.1–1.2). For a monomer ratio of 10, the initiation efficiency observed for α-CD and γ-CD, around 86–89%, is lower than that observed for β-CD. Peptu et al. also found superior performances for the β-CD in the CD-initiated ring-opening oligomerization of lactide [22].

The reactions were extended to methylated CDs, i.e., 2,6-DMCD and 2,3-DMCD (entries 11 and 12, respectively, structures given in Figure 2). Each of these CDs only bears 7 hydroxyl groups compared to the native CD, primary alcohols for the former and secondary alcohols for the latter. The polymerization was found to be quantitative for both partially methylated CDs. In the case of 2,3-DMCD, the C-6 primary hydroxyl groups could initiate quantitatively the polymerization while the C-3 secondary hydroxyl groups could only partially initiate in the case of 2,6-DMCD. The secondary nature of the hydroxyl group together with a possible steric hindrance provided by the two methyl substituents leads to a lower initiation rate than the primary alcohol, with a substantial propagation rate in both cases.

If 2,6-dimethylated-β-CD has already been reported as an initiator for the ring-opening polymerization of lactide in the presence of tin octanoate [31], this is the first time, to the best of our knowledge, that a seven-arm β-CD-polylactide conjugate has been formed starting from 2,3-dimethylated-β-CD. In addition, we are also not aware of fully functionalized α-CD and γ-CD polylactide conjugates, with 18 and 24 arms, respectively.

Finally, we performed the ^1^H decoupled NMR of several samples (given in the SI Section) to determine the tacticity of the polylactides formed, and they were found to be typical of atactic poly(*rac*-lactide) (see e.g, ref. [32]).

## 3. Materials and Methods

### 3.1. Materials

*Rac*-lactide (*rac*-LA) and 4-dimethylaminopyridine (DMAP) were purchased from Sigma-Aldrich (Germany) and co-evaporated three times with toluene, followed by sublimation under vacuum at 85 °C and storage in a glovebox. Native α-, β- and γ-cyclodextrins (CDs) and heptakis-(2,6-di-*O*-methyl)-β-cyclodextrin (2,6-DMCD) were purchased from Sigma-Aldrich (Germany) and were co-evaporated three times with toluene. Deuterated solvents (DMSO-*d_6_* and D_2_O) were purchased from Euriso-Top (France). Adamantane was obtained from Sigma-Aldrich (Germany) and was purified by crystallization from acetone and then dried under vacuum. All reagents and anhydrous solvents used for the synthesis of the heptakis-(2,3-di-*O*-methyl)-β-cyclodextrin (2,3-DMCD) were purchased from Sigma-Aldrich (Germany) and used directly without any further purification. Anhydrous toluene was taken from a solvent purification system (MBraun MB SPS-800). All the experiments were prepared in a glovebox.

### 3.2. Synthesis

#### 3.2.1. Preparation of Heptakis-(2^I–VII^,3^I–VII^-di-*O*-methyl)-β-Cyclodextrin (2,3-DMCD)

Heptakis-(2^I–VII^,3^I–VII^-di-*O*-methyl)-β-cyclodextrin (2,3-DMCD) was obtained in three steps from the native β-cyclodextrin using the usual method as already described in the literature. Analytical data were identical to the literature [33].

#### 3.2.2. General Procedure for *Rac*-Lactide (LA) Polymerization Initiated by CD

All initiators were dried several days at room temperature under ultra-high vacuum (5 × 10^−6^ mbar) before use as it was done in previous studies [15,23]. In a typical polymerization run, purified *rac*-LA (ca. 300 mg), initiator (native or *O*-methylated CD or inclusion complexes) ([*rac*-LA]/[ROH] = 2–30) and purified DMAP ([DMAP]/[ROH] = 2) were weighed into a glass reactor in a glovebox. The reactor was sealed, taken out from the glovebox and put in an oil bath at 120 °C. The starting time was considered when the lactide was molten. The reaction mixture was magnetically stirred (300 rpm) under an inert atmosphere for a given time (usually 10–60 min until the stirring bar stopped stirring). At the end of the reaction, a small quantity of dichloromethane was added to dissolve the solid product. A sample of dichloromethane solution was taken for ^1^H-NMR analysis of the crude product to determine the monomer conversion. In order to remove the unreacted monomer and catalyst, the remaining solution was precipitated in cold diethyl ether (DP < 10) or methanol (DP > 10), decanted or filtered to give a white powder dried overnight under vacuum at room temperature. A sample of the final product was analyzed by ^1^H-NMR to determine the degree of polymerization and by SEC to determine the dispersity.

PLA: ^1^H NMR (300.13 MHz, DMSO-*d_6_*) δ (ppm) 5.21 (m, -OC*H*(CH_3_)CO- PLA); 4.22 (q, COOC*H*(CH_3_)-OH PLA end group); 1.46 (d, *J* = 7.0 Hz, -OCH(C*H*_3_)CO- PLA); 1.29 (d, *J* = 6.9 Hz, 3H, -COOCH(C*H*_3_)-OH PLA end group).

### 3.3. Characterization of the PLA Functionalized CD

#### 3.3.1. Nuclear Magnetic Resonance (NMR) Analysis

^1^H NMR spectra were recorded with a Bruker AVANCE III HD 300 spectrometer at 300.13 MHz (7.05 Tesla) in DMSO-*d_6_* at 298 K. Approximately 3 mg of sample was directly dissolved into the NMR tube in 0.6 mL of solvent for ^1^H. Chemical shifts (ppm) are given in *δ*-units and were calibrated using the residual resonances of the solvent. Data acquisition and analysis were performed using Bruker TopSpin 3.2. The conversion (Conv.) and degree of polymerization (DP) were calculated by the integration of the protons of the C*H* group of *rac*-LA, the proton of the C*H* group of polylactide and the protons of the C*H*-OH end group of polylactide (PLA), which are at *δ* = 5.46, 5.21 and 4.22 ppm in DMSO-*d_6_*, respectively. The conversion was calculated from the ratio C*H* PLA/(C*H* PLA + C*H rac*-lactide). The degree of polymerization was determined from the ratio C*H* PLA/2C*H*-OH. The calculated degree of polymerization is equal to the ratio of the *rac*-lactide/hydroxyl multiplied by the conversion. Homonuclear decoupled ^1^H NMR spectra were recorded on an Avance NEO 400 Bruker spectrometer (9.4 Tesla) regulated at 300 °K in CDCl_3_. The coupling effect between the methine and the CH_3_ proton of polylactide was removed by irradiation of the CH_3_ area (1.58 ppm or 1.54 ppm) under O2 (635 Hz or 617 Hz sample dependent, PLW24 = 0.00068 Watt).

#### 3.3.2. Size Exclusion Chromatography (SEC) Analysis

Size exclusion chromatography (SEC) was performed at a concentration of 2 g/L in THF as eluent at 40 °C using a Waters SIS HPLC-pump, a Water 2414 refractometer and Water Styragel column HR3 and HR4. The calibration was performed using polystyrene standards (M_w_ 820, 2727, 4075, 12,860, 32,660, 45,730, 95,800, 184,200, 401,340 and 641,340 g/mol). The dispersity (D_M_) was determined by SEC.

### 3.4. Synthesis and Characterization of Inclusion Complexes

#### 3.4.1. General Procedure for Formation of Inclusion Complexes (DMAP/β-CD or Adamantane/β-CD)

**Figure 9:**
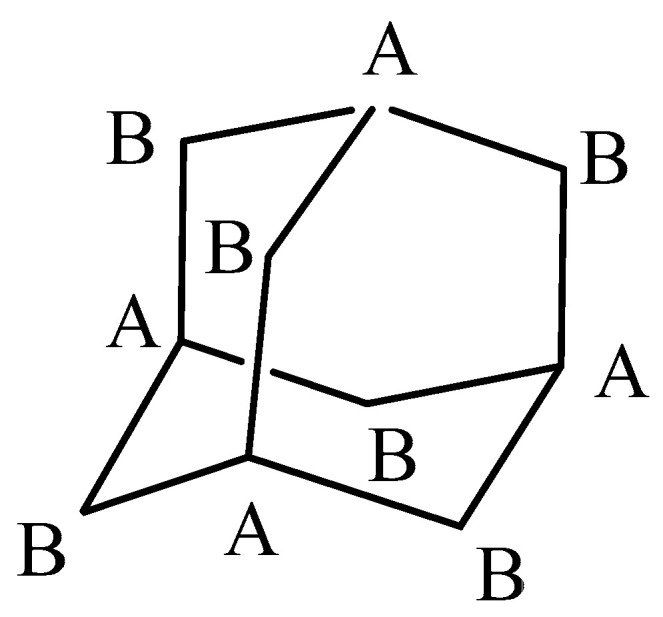
Adamantane structure.

A mixture of β-CD (1 mmol) and DMAP or adamantane (structure given in Figure 9) (1 mmol) was allowed to form a complex in an aqueous solution (30 mL) with stirring at 30 °C for 5 h. The solution was slowly cooled to 0 °C and the precipitate formed was filtered to give white powder. The crude product was recrystallized in aqueous solution, purified from water and dried under high vacuum at room temperature to obtain a pure white sample. Yield: 60% (DMAP) and 55% (adamantane).

- DMAP/β-CD Inclusion Complex (see Figure S2 in SI)

^1^H NMR (300.13 MHz, DMSO-*d_6_*) δ (ppm) 8.09 (dd, 2H, *J* = 1.6 and 4.9 Hz, *H*_aromDMAP_); 6.58 (dd, 2H, *J* = 1.6 and 4.9 Hz, *H*_aromDMAP_); 5.76 (d, 7H, *J* = 6.8 Hz, O_2CD_*H*); 5.70 (d, 7H, *J* = 2.3 Hz, O_3CD_*H*); 4.83 (d, 7H, *J* = 3.4 Hz, *H*^I-VII^_1CD_); 4.48 (t, 7H, *J* = 5.9 Hz, O_6CD_*H*); 3.73–3.50 (m, 28H, *H*^I-VII^_3CD_, *H*^I-VII^_5CD_ and *H*^I-VII^_6CD_); 3.41–3.24 (m, 14H, *H*^I-VII^_2CD_ and *H*^I-VII^_4CD_); 2.94 (s, 6H, C*H*_3DMAP_).

- Adamantane/β-CD Inclusion Complex (see Figure S3 in SI)

^1^H NMR (300.13 MHz, DMSO-*d_6_*) δ (ppm) 5.65 (m, 14H, O_2CD_*H* and O_3CD_*H*); 4.83 (d, 7H, *J* = 3.7 Hz, *H*^I-VII^_1CD_); 4.42 (t, 7H, *J* = 5.9 Hz, O_6CD_*H*); 3.70–3.50 (m, 28H, *H*^I-VII^_3CD_, *H*^I-VII^_5CD_ and *H*^I-VII^_6CD_); 3.40–3.24 (m, 14H, *H*^I-VII^_2CD_ and H^I-VII^_4CD_,); 1.87 (s, 4H, C_A_*H_Ada_*); 1.72 (m, 12H, C_B_*H*_2*Ada*_).

#### 3.4.2. Determination of the DMAP/β-CD Inclusion Complex Stoichiometry by Job Plot Method

In order to determine the stoichiometry of the complex between DMAP and β-CD (establishment of Job curves), two solutions of 10 mM in deuterium oxide (D_2_O) of each component were prepared. Different NMR tubes were prepared by mixing the equimolar solutions to a constant volume (0.6 mL) and varying the ratio DMAP/β-CD to describe a range of the concentration fractions between 0 < r < 1 (r = [X]/([H] + [G]), where X = H or G and [H] and [G] were, respectively, the concentration of host (β-CD) and guest (DMAP) in the complex sample. The total concentration [H] + [G] = 10 mM was kept constant for each analysis. ^1^H NMR spectra were recorded on a Bruker AVANCE III HD 300 spectrometer at 298 K (number of scans = 16, delay of relaxation = 3 s).

#### 3.4.3. Characterization of the DMAP/β-CD Inclusion Complex Geometry

The geometry of the inclusion complex was finally characterized by NMR in deuterium oxide (D_2_O) at 300 K. NMR spectra were recorded on a Bruker AVANCE NEO 400 spectrometer operating at 400.33 MHz. Chemical shifts were measured relative to the residual D_2_O signal at 4.79 ppm. 1D spectra were collected recording 8 scans. The 2D Rotating-frame Overhauser Effect Spectroscopy (ROESY) spectrum was acquired in the phase-sensitive mode with the same spectrometer and Bruker standard parameters (pulse program roesyadjsphpr) using a TBI probe. Each spectrum consisted of a matrix of 2048 (F2) by 256 (F1) points covering a width of 4000 Hz.

#### 3.4.4. Estimation of the Association Constant (K_a_) of DMAP/β-CD Inclusion Complex

Approximations of the association constant *K*_a_ and Δδ_c_ (chemical shift difference between the free and completely complexed substrate) were determined from the Benesi–Hildebrand method via the following Equation (1):

1/Δδ_obs_ = 1/(*K*_a_ * Δδ_c_ *[β-CD]) + 1/Δδ_c_ (1)

where Δδ_obs_ (defined by δ_free_ − δ_obs_) was determined by ^1^H NMR. Different NMR tubes in deuterium oxide (D_2_O) were prepared maintaining the DMAP concentration constant (1 mM) to form the complex with β-CD and varying the β-CD concentration (from 5.5 to 15.5 mM). ^1^H NMR spectra were recorded on a Bruker AVANCE III HD 300 spectrometer at 298 K (number of scans = 16, delay of relaxation = 3 s).

## 4. Conclusions

A DMAP/β-CD inclusion complex has been formed and isolated for the first time, and thoroughly characterized by NMR. We have shown using 2D ROESY NMR that the inclusion of DMAP into the cavity takes place through the secondary side of the β-CD. We also determined that the complex is of 1:1 stoichiometry and is characterized by an association constant of 88.2 M^−1^. Its use as an initiator for the ring-opening polymerization of *rac*-lactide catalyzed by DMAP (DMAP/ROH = 2) led to quantitative polymerizations, as observed using the native β-CD as the initiator, but to a partial initiation efficiency, providing more hydrophilic cyclodextrin-polylactyide conjugates. This may be ascribed to an interaction of the hydroxyl groups with the included DMAP or a steric hindrance induced by the proximity of the DMAP. If quantitative functionalization is also reached for native α- and γ-CDs as well as 2,3-dimethyl-cyclodextrin, the polymerization conducted using 2,6-dimethyl-cyclodextrin as the initiator also led to a partial initiation efficiency, which may be attributed to a lower reactivity of secondary alcohols.

## Figures and Tables

**Table 1:** Chemical shifts of DMAP (guest) and β-CD (host) protons in the free and complex states (1/1).

Proton	δ_free_ (ppm)	δ_c_ (ppm)	Δδ_c_^1^ (ppm)
H-1	5.0472	5.0356	0.012
H-2	3.6237	3.611	0.013
H-3	3.9446	3.868	**0.077**
H-4	3.5611	3.5583	0.003
H-5	3.8175	3.7086	**0.109**
H-6	3.8572	3.8368	**0.021**
H-a	6.674	6.6371	**0.037**
H-b	8.0420	8.0478	−0.006
H-c	2.9921	3.0078	−0.016

^1^ Δδ_c_ = δ_free_ − δ_c_ values were obtained as a result of the ^1^H NMR study (in D_2_O, 300 MHz, 300 K, D1 = 3, NS = 16).

**Table 2:** Ring-opening polymerization of *rac*-lactide at 120 °C in bulk using DMAP as a catalyst and various cyclodextrin-based initiators (DMAP/ROH = 2).

Entry	Init. ^1^	M/ROH	Time (min)	Conv. ^2^ (%)	DP/OH ^3^ Calc.	DP/OH ^4^ Final	Init Eff. ^5^ %	D_M_^6^
1 ^7^	DIC	30	60	4	-	-	-	
2	DIC	10	30	94	9.4	14	67	1.10
3 ^8^	β-CD	10	30	97	9.7	10	97	1.09
4	DIC	2	10	83	1.65	2.7	61	1.13
5	DIC	20	60	96	19.2	24.1	80	1.14
6	AIC	2	10	47	0.95	1.2	79	1.14
7	α-CD	10	10	97	9.6	11.2	86	1.07
8	α-CD	30	20	99	19.8	20.3	98	1.09
9	γ-CD	2	10	97	1.9	2.2	88	1.18
10	γ-CD	10	30	96	9.6	10.8	89	1.09
11	2,6-DM	30	60	99	29.8	*ca.* 80	37	1.49
12	2,3-DM	10	30	96	9.6	9.6	100	1.34

^1^ DIC = DMAP/β-CD inclusion complex, AIC = adamantane/β-CD inclusion complex. ^2^ Conversion determined by ^1^H NMR (see experimental section). ^3^ Number-average degree of polymerization per initiating hydroxyl group calculated considering the growth of one macromolecular chain per hydroxyl group. ^4^ Number-average degree of polymerization per initiating hydroxyl group measured by ^1^H NMR (see experimental section). ^5^ Relative amount (%) of the cyclodextrin OH groups that initiate the growth of a macromolecular chain calculated as follows: (DP/OH calc)/(DP/OH final)*100. ^6^ Dispersity measured by size exclusion chromatography (THF, 40 °C, PS standards, chromatograms given in the SI Section Figures S4–S13). ^7^ Blank experiment conducted with the sole DMAP/β-CD inclusion complex, without additional DMAP catalyst. ^8^ Taken from ref [23].

## Data Availability

Not applicable.

## Supplementary Materials

The following supporting information can be downloaded at online. Figure S1: Superposition of ^1^H NMR spectra of free DMAP (A), free β-CD (B) and a mixture (1:1) DMAP/β-CD (C) in D_2_O (3.7 mM, 400 MHz, 300 K), Figure S2: ^1^H NMR spectrum of DMAP/β-CD inclusion complex in DMSO-*d_6_* (300 MHz, 298 K), Figure S3:^1^H NMR spectrum of Adamantane/β-CD inclusion complex in DMSO-*d_6_* (300 MHz, 298 K), Figure S4: SEC chromatogram (THF, 40 °C, polystyrene standards) of entry 2, Figure S5: SEC chromatogram (THF, 40 °C, polystyrene standards) of entry 4, Figure S6: SEC chromatogram (THF, 40 °C, polystyrene standards) of entry 5, Figure S7: SEC chromatogram (THF, 40 °C, polystyrene standards) of entry 6, Figure S8 SEC chromatogram (THF, 40 °C, polystyrene standards) of entry 7:, Figure S9: SEC chromatogram (THF, 40 °C, polystyrene standards) of entry 8, Figure S10: SEC chromatogram (THF, 40 °C, polystyrene standards) of entry 9, Figure S11: SEC chromatogram (THF, 40 °C, polystyrene standards) of entry 10, Figure S12: SEC chromatogram (THF, 40 °C, polystyrene standards) of entry 11, Figure S13: SEC chromatogram (THF, 40 °C, polystyrene standards) of entry 12, Figure S14: ^1^H Homonuclear decoupled NMR of Entry 2, Figure S15: ^1^H Homonuclear decoupled NMR of Entry 5, Figure S16: ^1^H Homonuclear decoupled NMR of Entry 10, Figure S17: ^1^H Homonuclear decoupled NMR of Entry 11.
