# Supplementary material for: Cyclodextrins Initiated Ring-Opening Polymerization of Lactide Using 4-Dimethylaminopyridine (DMAP) as Catalyst: Study of DMAP/β-CD Inclusion Complex and Access to New Structures
Source: Molecules. 2022 Feb 6;27(3):1083. doi: 10.3390/molecules27031083 (PMC8838909; doi:10.3390/molecules27031083)
Supplement: Supplementary file 1 [file molecules-27-01083-s001.zip › molecules-1574488-supplementary.pdf]

Supporting Informations

Cyclodextrins initiated ring-opening polymerization of lactide using 4-dimethylaminopyridine (DMAP) as catalyst: study of DMAP/β-CD inclusion complex and access to new structures

Julie Meimoun [1], Yupin Phuphuak [1,2], Remi Miyamachi [1], Yong Miao [1], Marc Bria [3], Cyril Rousseau [4], Guilherme Nogueira [1,5], Andreia Valente [5], Audrey Favrelle-Huret [1,*] and Philippe Zinck [1,*]

[1] Univ. Lille, CNRS, Centrale Lille, Univ. Artois, UMR 8181—UCCS—Unité de Catalyse et Chimie du Solide, F-59000 Lille, France; julie.meimoun90@gmail.com (J.M.); yupinp@nu.ac.th (Y.P.); cherry.milky.853@gmail.com (R.M.); Yong.Miao@gnubiotics.com (Y.M.);
guilherme.nogueira91@gmail.com (G.N.)

[2] Department of Chemistry, Faculty of Science, Naresuan University, Phitsanulok 65000, Thailand

[3] Univ. Lille, CNRS, INRA, Centrale Lille, ENSCL, Univ. Artois, FR 2638—IMEC—Institut Michel-Eugène Chevreul, Pôle RMN, F-59000 Lille, France; marc.bria@univ-lille.fr

[4] Univ. Artois, CNRS, Centrale Lille, ENSCL, Univ. Lille, UMR 8181—UCCS—Unité de Catalyse et Chimie du Solide, F-62300 Lens, France; cyril.rousseau@univ-artois.fr

[5] Centro de Química Estrutural, Institute of Molecular Sciences and Departamento de Química e Bioquímica, Faculdade de Ciências, Universidade de Lisboa, Campo Grande, 1749-016 Lisboa, Portugal; amvalente@fc.ul.pt

* Correspondence: audrey.huret@univ-lille.fr (A.F.-H.); philippe.zinck@univ-lille.fr (P.Z.)

Nuclear Magnetic Resonance (NMR) analysis

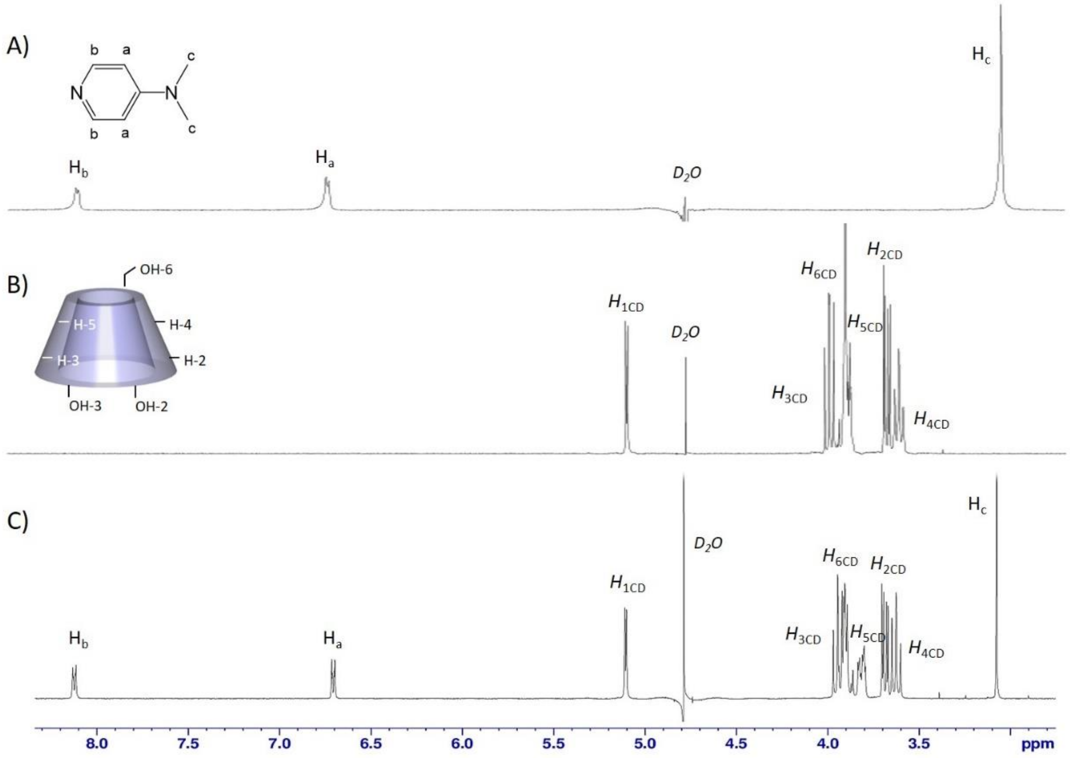

Figure S1. Superposition of ^{1}H NMR spectra of free DMAP (A), free β-CD (B) and a mixture (1:1) DMAP/β-CD (C) in D_2O (3.7 mM, 400 MHz, 300 K)

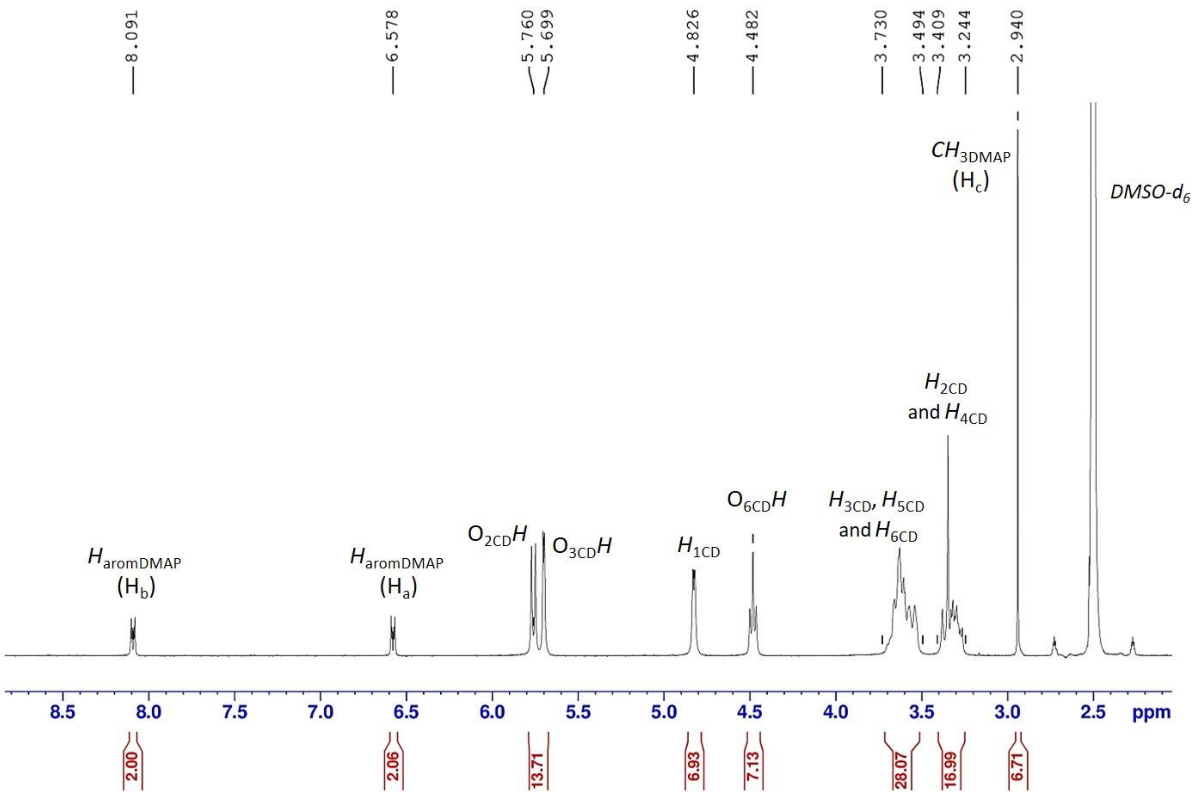

Figure S2. ^{1}H NMR spectrum of DMAP/β-CD inclusion complex in DMSO-d_6 (300 MHz, 298 K)

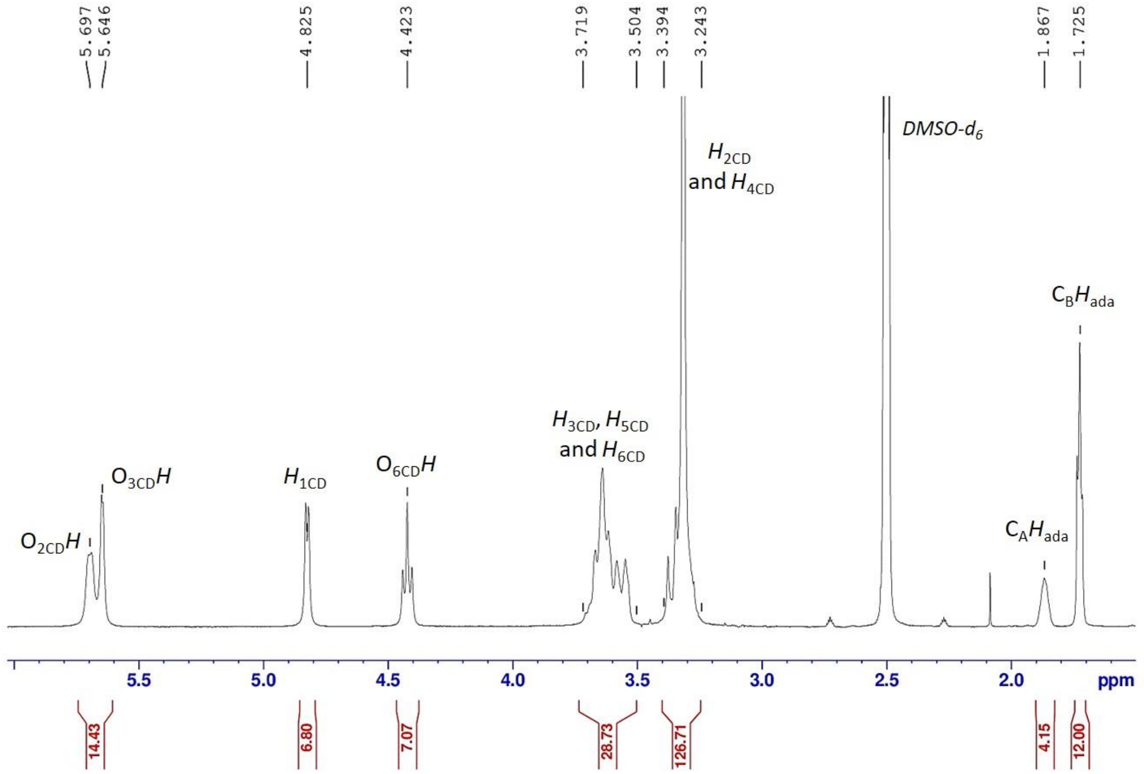

Figure S3. ^{1}H NMR spectrum of Adamantane/β-CD inclusion complex in DMSO-d_6 (300 MHz, 298 K)

Size Exclusion Chromatography (SEC) analysis

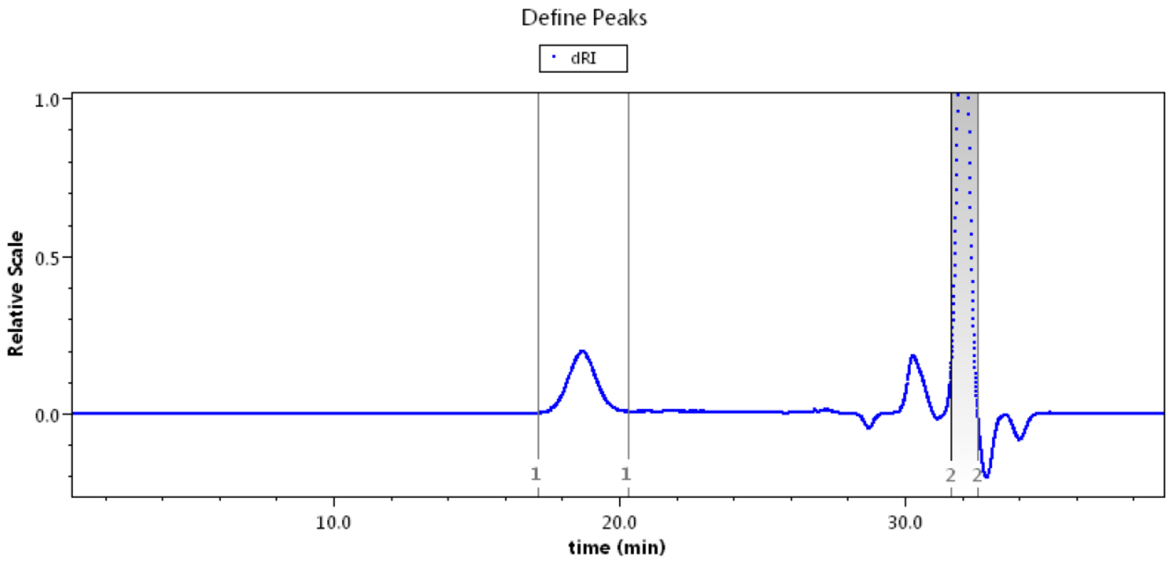

Figure S4. SEC chromatogram (THF, 40°C, polystyrene standards) of entry 2

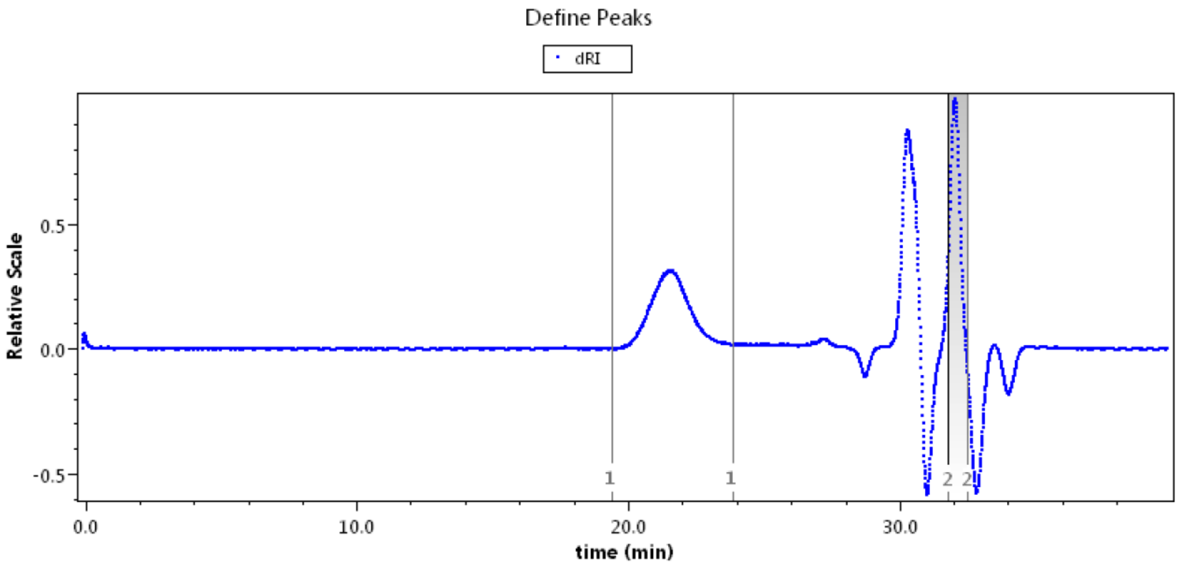

Figure S5. SEC chromatogram (THF, 40°C, polystyrene standards) of entry 4

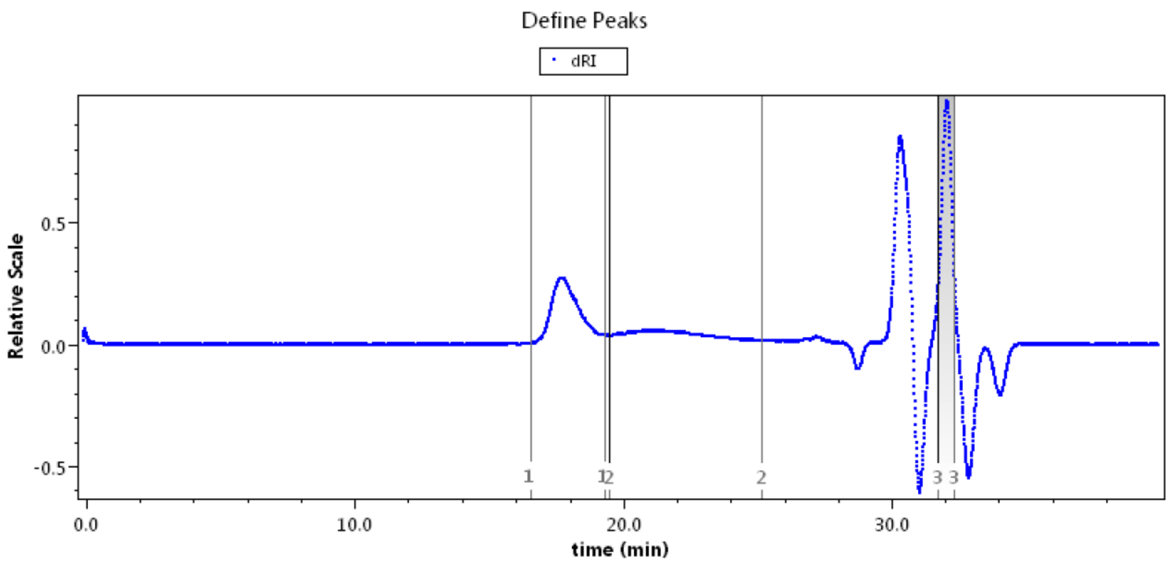

Figure S6. SEC chromatogram (THF, 40°C, polystyrene standards) of entry 5

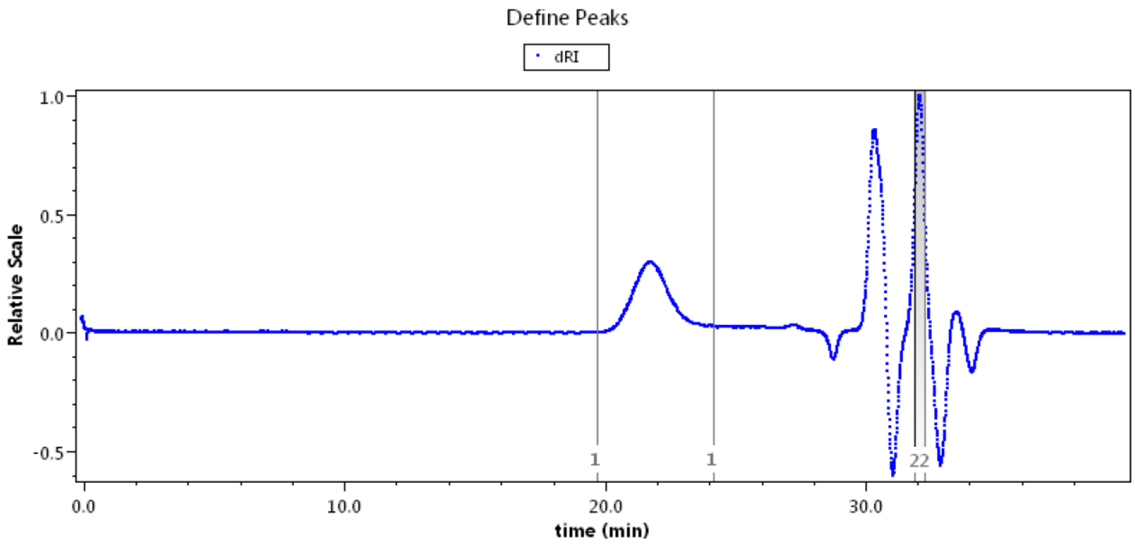

Figure S7. SEC chromatogram (THF, 40°C, polystyrene standards) of entry 6

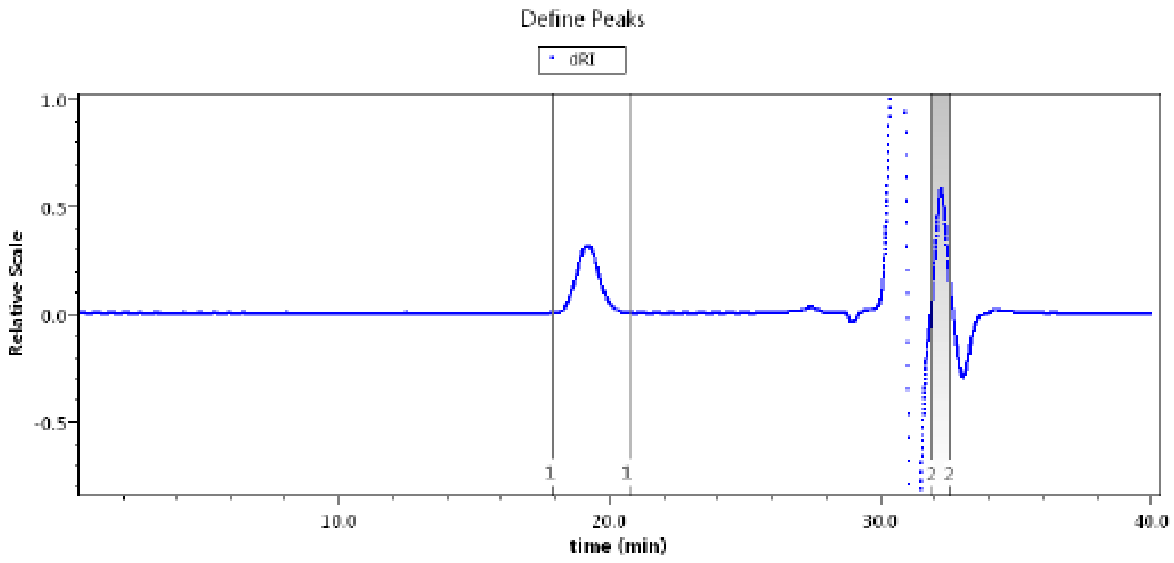

Figure S8. SEC chromatogram (THF, 40°C, polystyrene standards) of entry 7

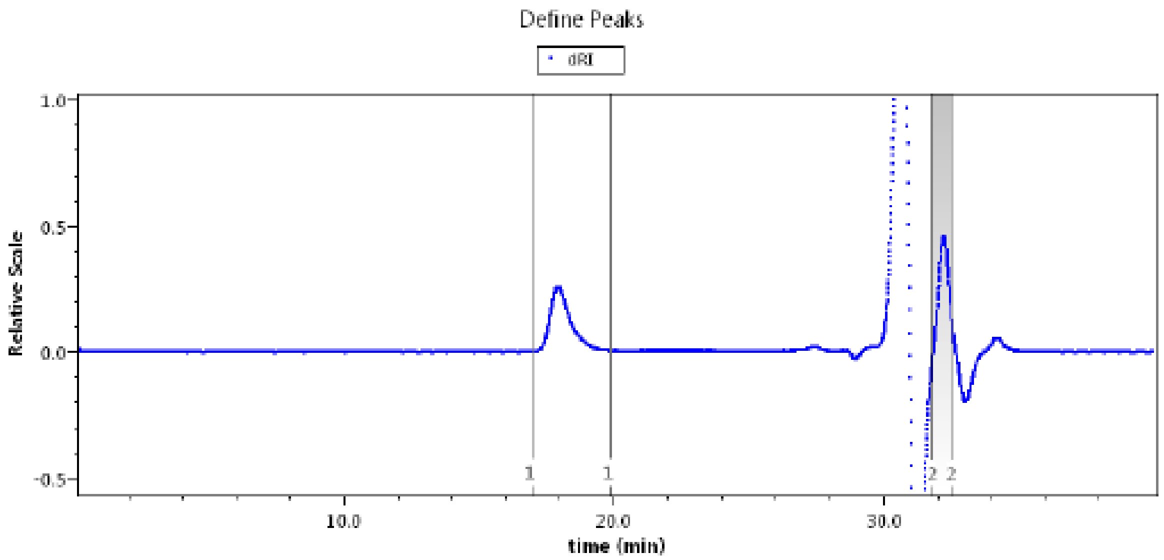

Figure S9. SEC chromatogram (THF, 40°C, polystyrene standards) of entry 8

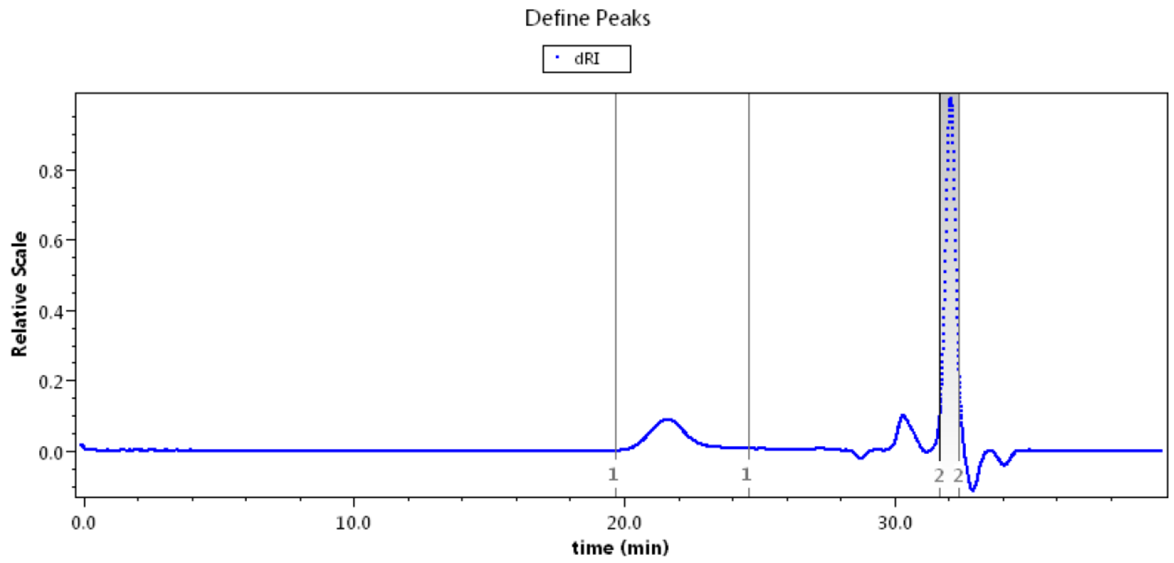

Figure S10. SEC chromatogram (THF, 40°C, polystyrene standards) of entry 9

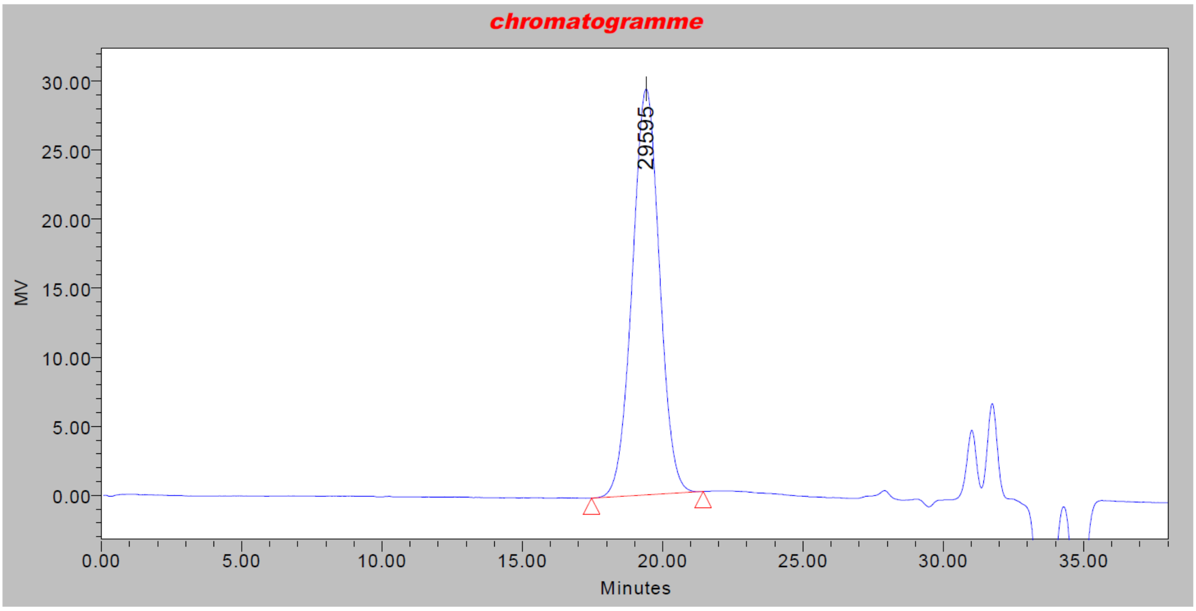

Figure S11. SEC chromatogram (THF, 40°C, polystyrene standards) of entry 10

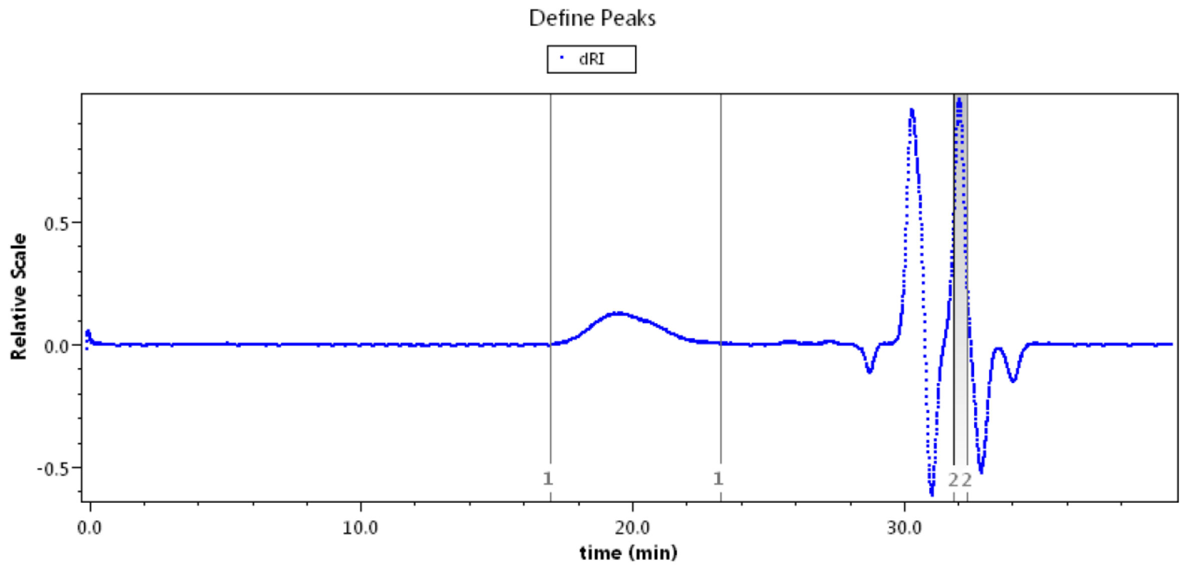

Figure S12. SEC chromatogram (THF, 40°C, polystyrene standards) of entry 11

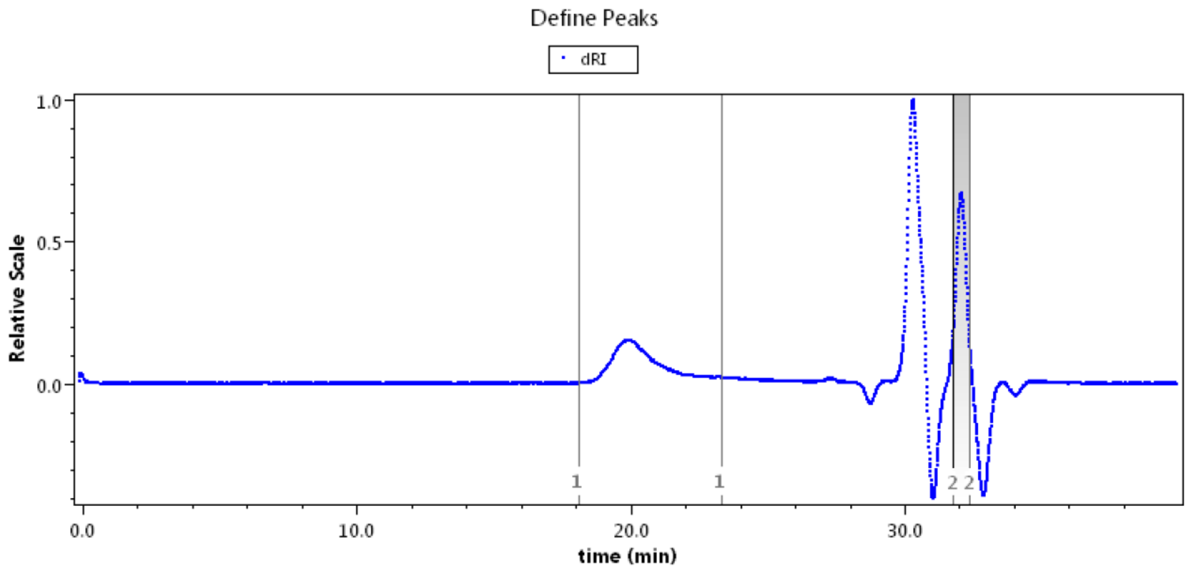

Figure S13. SEC chromatogram (THF, 40°C, polystyrene standards) of entry 12

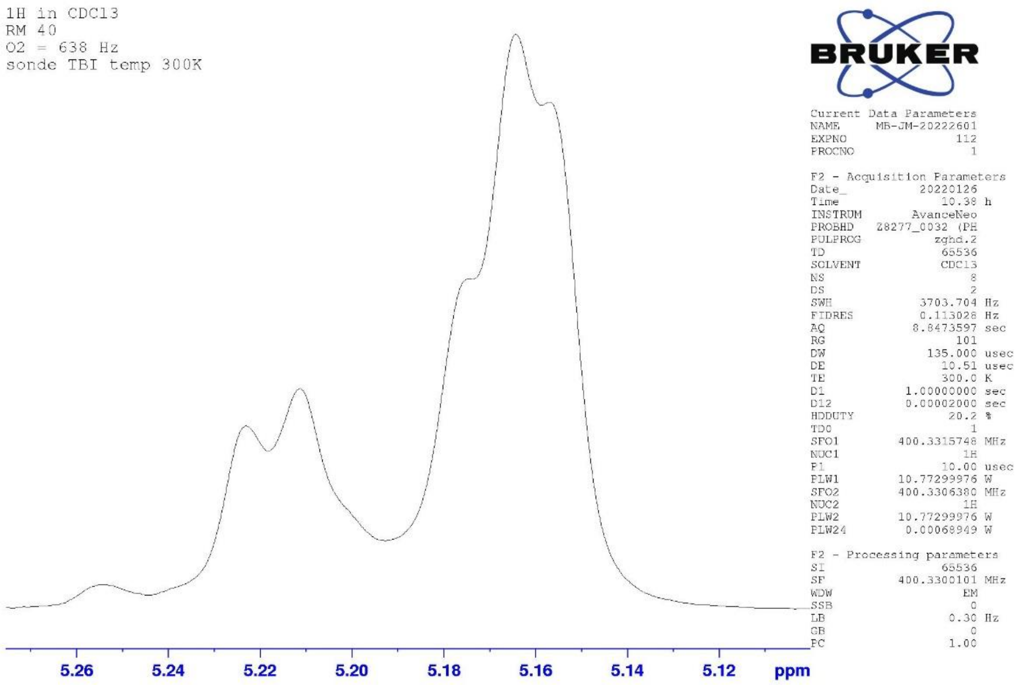

Figure S14. ^{1}H Homonuclear decoupled NMR of Entry 2

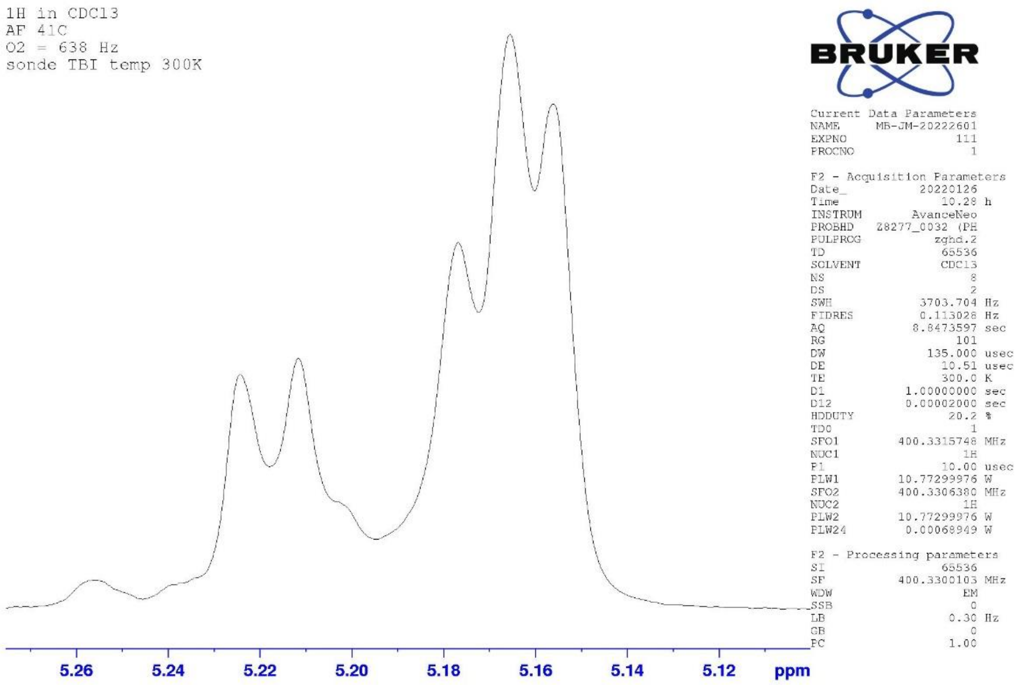

Figure S15. ^{1}H Homonuclear decoupled NMR of Entry 5

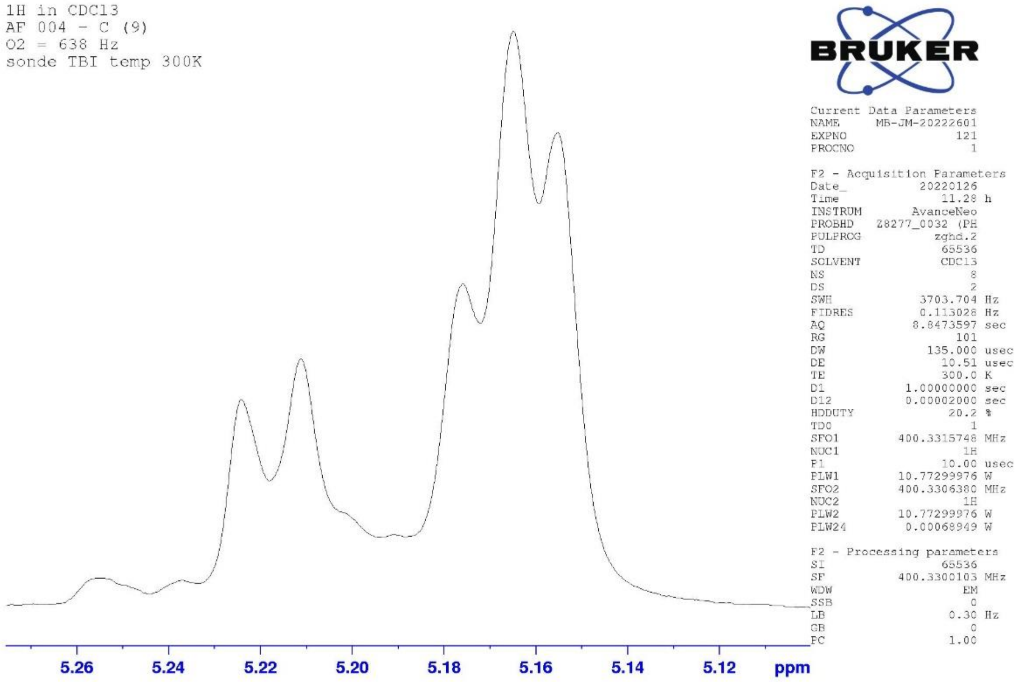

Figure S16. ^{1}H Homonuclear decoupled NMR of Entry 10

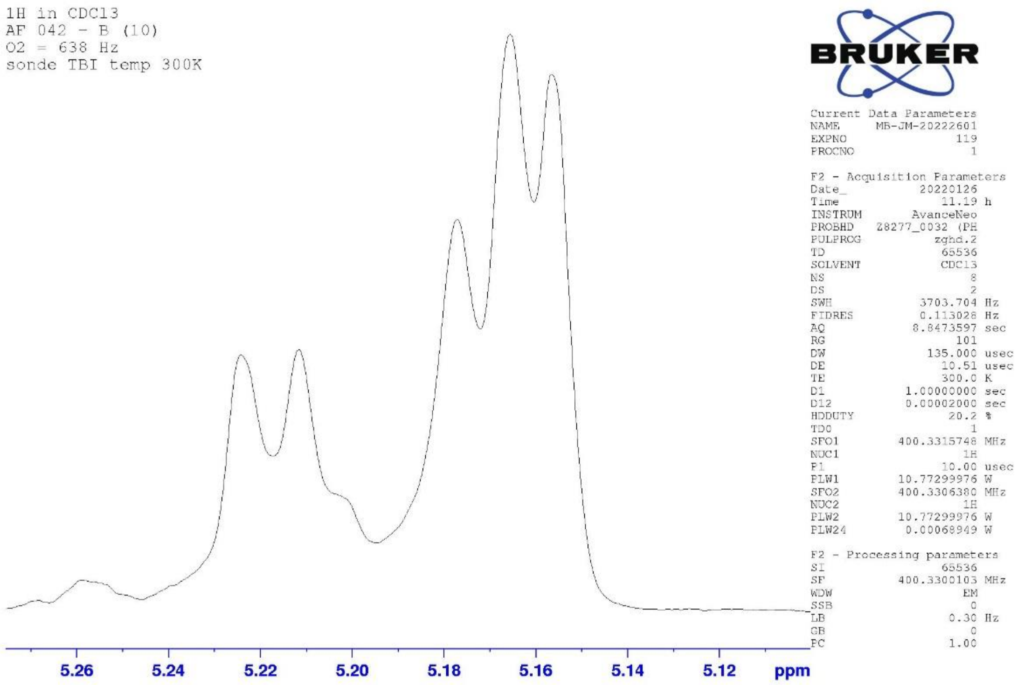

Figure S17. ^{1}H Homonuclear decoupled NMR of Entry 11
